# Complexity and Target Preservation in Category Maps

**DOI:** 10.3390/e28070796

**Published:** 2026-07-13

**Authors:** Christoph D. Dahl

**Affiliations:** Graduate Institute of Mind, Brain and Consciousness, Taipei Medical University, New Taipei City 235, Taiwan; christoph.d.dahl@gmail.com

**Keywords:** categorisation, entropy, mutual information, information bottleneck, category learning, visual cognition, representation learning, neural networks, nuisance variation

## Abstract

Categorisation is often treated as a form of compression: a high-dimensional stimulus space is reduced to a smaller set of behaviourally or cognitively useful classes. However, compression alone does not determine whether a category map is useful. The present manuscript develops an information-theoretic framework for evaluating categorisation in terms of both category complexity and target-relevant information preservation. Here, categorisation is treated as a many-to-one mapping from stimulus instances to category labels, and category entropy quantifies the distribution of the resulting labels. Across a set of synthetic demonstrations, alternative category maps over the same stimulus space are shown to preserve different target variables, including identity, action, nuisance, and hierarchical category structure. The framework is then extended to learned visual representations by analysing layer-derived category maps from a pretrained ResNet-50 network applied to CIFAR-10 images. Clean-only, strong- and mild-nuisance controls test whether layer-derived maps preserve object or nuisance information within nuisance conditions. The results show that category maps can have substantial entropy while preserving information about a variable that is not aligned with the specified target and that the value of a categorisation depends on the target variable to be preserved. The manuscript argues that categorisation should therefore be evaluated not only by compression or separability, but by the information retained about a specified cognitive, behavioural, or computational target.

## 1. Introduction

Categorisation is one of the central operations of cognition [1,2]. Organisms and artificial systems encounter a world whose perceptual states are highly variable, continuous, and often noisy. Nevertheless, behaviour rarely depends on every distinguishable detail of that world. Different visual views can be treated as the same object, different individuals can be treated as members of the same kind, and different objects can be grouped according to similar consequences for action. In this broad sense, categorisation compresses the stimulus space: many possible sensory states are mapped onto a smaller number of classes. This compression view is intuitively attractive, but it is incomplete. A compression can be simple without being useful. A stimulus set can be compressed into one category, into arbitrary clusters, or into categories based on irrelevant features. Such category maps reduce complexity, yet they may preserve little information about what the system must recognise, predict, or control. Conversely, a more complex category map may be useful if it preserves a variable that is relevant for behaviour or inference [3,4,5]. Thus, the relevant question is not whether categorisation reduces stimulus complexity, but which target-relevant information remains available after that reduction.

The importance of this distinction is visible in classical work on categories and concepts, but the connection made here is operational rather than comprehensive. Natural categories are structured by family resemblance, typicality, and category-level regularities that support inductive generalisation [2,6,7,8]. The basic-level category literature is particularly relevant because it treats category level as a trade-off between informativeness and economy: a useful category preserves predictive regularities while avoiding unnecessary detail [3,4,7]. From the present perspective, this can be reformulated as a complexity–relevance problem. Different category levels impose different reductions on the same stimulus space, and their usefulness depends on which target variables are preserved. This target-relative view is also compatible with exemplar, rational, and computational accounts in which categories support generalisation by encoding assumptions about which properties should transfer across instances [5,9,10,11,12]. The present manuscript does not model category learning, similarity judgement, or conceptual coherence. This literature motivates only the diagnostic question of whether a candidate category map preserves information about a specified target.

Information theory provides a natural language for this issue. Since the work of Shannon [13], entropy has provided a formal measure of uncertainty, and mutual information has quantified how much uncertainty about one variable is reduced by observing another [14]. In sensory coding, efficient coding theories have long treated perceptual systems as transformations that reduce redundancy while preserving behaviourally important information [15]. The information bottleneck framework makes this trade-off explicit by seeking compressed representations that retain information about a relevant target variable [16,17]. The present approach also builds on the more general idea that transformations can be analysed as information-funnelling operations, in which many initial states are compressed into fewer structured outcomes while entropy and residual state information are tracked across the transformation [18]. Related information-theoretic approaches have been used to analyse behavioural sequences, animal movement patterns, and spatial relations in collective behaviour [19,20,21]. In this context, the geometry of animal groups has also been analysed as an information-theoretic problem [22]. The central logic is directly applicable to categorisation. A category map should not be evaluated only by the entropy of the categories it produces. It should be evaluated by the information that those categories preserve about a target variable. This distinction is also relevant for contemporary representation learning. Deep neural networks transform images into hierarchical activation spaces in which object categories, textures, nuisance variables, and task-relevant distinctions may be represented to different degrees [23,24,25,26]. These models have become useful tools for comparing artificial and biological visual representations [27,28,29,30]. Yet the same caution applies: a low-dimensional or clustered representation is not necessarily a semantically useful one [31,32]. If the dominant structure in an activation space corresponds to blur, noise, or other nuisance variables, an unsupervised category map may preserve those variables more strongly than object identity. In such a case, the representation is compressed and structured but not useful for the intended semantic target.

The present manuscript develops this point in a deliberately simple formal setting. A category map is defined as a function that assigns each stimulus to a category. Its complexity is measured by the entropy of the induced category variable. Its relevance is measured by the mutual information between the category variable and a target variable. This formulation allows the same stimulus space to be analysed under different candidate target variables, such as identity, action, nuisance condition, or category level. It also allows different category maps to be compared even when they have similar complexity. Four synthetic demonstrations are used to establish the principle, followed by a fifth exploratory demonstration in learned visual representations. First, several category maps are compared over the same two-dimensional stimulus space, showing that category entropy alone cannot distinguish useful from useless partitions. Second, the same category maps are evaluated with respect to multiple target variables, showing that a category map can be useful for one target and useless for another. Third, robustness under perturbation is examined, showing how preserved information changes when sensory variation increases. Fourth, hierarchical category maps are analysed, showing how subordinate, basic, and broader action/superordinate-like category maps can be interpreted as different compressions of the same stimulus space. A fifth, exploratory demonstration extends the analysis to a pretrained visual neural network. CIFAR-10 images are passed through ResNet-50, activation spaces are clustered at selected layers, and the resulting layer-derived category maps are evaluated against object-relevant and nuisance-relevant targets. Additional conditional controls ask whether object information remains available within nuisance conditions, and a supervised-readout analysis asks whether target information can be recovered when the readout objective is specified explicitly. This extension is not intended as a full theory of deep-network categorisation. Its purpose is narrower: to show that the same information-theoretic problem arises in learned representations. A non-trivial category map can preserve substantial information, but that information may concern a nuisance variable rather than the semantic target of interest. Conversely, target information may remain available to a supervised readout even when it is not the dominant structure recovered by unsupervised clustering.

The present study makes three contributions. First, category complexity is separated from target preservation by treating a category map as a compression whose complexity is measured by category entropy, $H(C_{m})$, and whose usefulness is measured by the information it preserves about a specified target, $I(C_{m};T)$. Second, this logic is generalised beyond a single target by evaluating the same category map against identity-like, action-like, nuisance, geometric, hierarchical, and object-relevant variables. This shows that a category map is not intrinsically useful or useless. Its value depends on which target variable *T* is to be preserved. Third, the same problem is shown to arise in learned visual representations. Unsupervised activation-space partitions can preserve substantial information about nuisance conditions, even when the object class is the intended semantic target. Conditional object information, $I(C_{m};Y_{\mathrm{obj}}|V)$, is therefore used to ask whether object information remains available within nuisance conditions, where *V* denotes image-transformation condition. A supervised-readout analysis then asks a complementary question: whether object or nuisance information can be recovered when the readout target is specified explicitly. The framework therefore extends compression-based and separability-based views by requiring the target variable of categorisation to be specified explicitly.

## 2. Methods

### 2.1. Overview of the Modelling Strategy

The analyses were designed to evaluate category maps as target-preserving compressions. A category map was treated as a transformation from a stimulus space to a category variable. The central question was not whether a transformation reduced the complexity of the stimulus space, but whether the resulting categories preserved information about a specified target variable. This logic follows the general information-theoretic distinction between compression and preserved target information [13,14,16,17]. This logic was applied in two stages. First, synthetic stimulus spaces with known generative structure were used to establish the formal relations among category entropy, target information, robustness, and classification level. Second, the same information-theoretic analysis was applied to layer-derived category maps from a pretrained visual neural network. The synthetic demonstrations were deliberately simple. Their purpose was to isolate the formal logic of target-preserving compression, not to model the full complexity of natural category learning. The neural network extension served a different role. It examined whether the same analysis could be applied to learned representations in a pretrained visual classifier [26,33], using CIFAR-10 images as a compact natural-image stimulus set [34]. The analysis asked whether unsupervised partitions of activation space preserved object-relevant information or information about nuisance conditions, defined here as the image-transformation state: clean, blurred, pixelated, or noise-perturbed. Table 1 summarises the five demonstrations, including their stimulus sources, candidate category maps, target variables, main information-theoretic quantities, and purpose.

### 2.2. Category Maps as Target-Preserving Compressions

Let *S* denote the stimulus variable, with individual stimulus instances s∈S, and let C=f(S) denote the category variable induced by a category map

(1) f:S→C.

Here, *C* is the category label assigned to each stimulus instance. The category map compresses the stimulus space whenever multiple distinct stimuli are assigned to the same category. All probabilities in the following expressions were estimated from empirical frequencies over the simulated or analysed samples. The complexity of the resulting category variable was quantified by category entropy,

(2) $$H\left(C\right)=-\sum_{c}p\left(c\right)\log_{2}p\left(c\right),$$

where p(c) denotes the probability of category *c*. Low entropy indicates a coarse or collapsed category map; high entropy indicates a richer set of category distinctions [13,14]. Category entropy alone does not specify what information is preserved by the category map. Therefore, each category map was evaluated relative to one or more target variables *T*. The target variable *T* denotes the variable whose preservation is being evaluated, such as identity, action relevance, nuisance condition, or hierarchical category level. Preserved target information was quantified by mutual information,

(3) $$I\left(C;T\right)=\sum_{c,t}p\left(c,t\right)\log_{2}\frac{p(c,t)}{p(c)p(t)}.$$

Here, p(c,t) denotes the joint probability of category *c* and target value *t*, and p(c) and p(t) denote the corresponding marginal probabilities. Equivalently,

(4) I(C;T)=H(T)−H(T∣C),

where H(T∣C) is the remaining uncertainty about the target after the category is known. For targets with different baseline entropies, preserved information was expressed as normalised mutual information,

(5) $$\mathrm{NMI}\left(C,T\right)=\frac{I(C;T)}{H(T)}.$$

This value measures the fraction of target uncertainty removed by the category map. A value of zero indicates that the category map preserves no information about the target. A value of one indicates that the target is fully determined by the category map. For target variables that may be confounded with nuisance structure, an additional conditional form was used. Let *V* denote a nuisance or transformation variable, such as image condition in the neural-network analyses. Conditional target preservation was quantified as

(6) $$\mathrm{NMI}\left(C,Y|V\right)=\frac{I(C;Y|V)}{H(Y|V)}.$$

This measure asks whether the category map *C* preserves information about the target *Y* within levels of the nuisance variable *V*. It therefore separates target information that remains available after accounting for nuisance structure from target information that may arise because target and nuisance variables are correlated in the sampled representation. Because mutual-information estimates can be positively biased in finite samples, permutation baselines were computed for the neural-network control analyses. This was relevant for comparisons across target variables such as $Y_{\mathrm{obj}}$ and *V*, because the empirical estimator can have different finite-sample baselines for targets with different numbers of cells and different marginal distributions. Normalising by target entropy places information values on a common fractional scale, but it does not by itself show that the empirical baselines are matched across targets. For unconditional target information, target labels were randomly permuted, and the corresponding normalised information was recomputed. For conditional target information, object labels were permuted within each nuisance condition, preserving the marginal nuisance structure while destroying the within-condition relationship between category labels and object identity. These permutation baselines therefore provide the relevant control: they preserve the empirical structure of each target comparison while removing the association between category labels and the target. The null-corrected values are interpreted relative to this target-specific finite-sample baseline. Null-corrected conditional target information was then defined by subtracting the mean within-nuisance permutation baseline from the observed conditional value. An illustrative quality score was also used to express the trade-off between target preservation and category complexity:

(7) $$Q_{T}=I\left(C;T\right)-\lambda H\left(C\right),$$

where λ controls the strength of the penalty on category complexity. For the illustrative quality-score panels, λ was fixed at 0.20. No conclusions depended on fitting or optimising this parameter. This score follows the same general logic as compression–relevance trade-offs in information-theoretic representation learning, where compressed variables are evaluated by the information they retain about a relevant target [16,17]. It was not treated as a unique normative measure.

### 2.3. Synthetic Demonstrations

#### 2.3.1. Stimulus Space and Target Variables

In the formal framework, *S* denotes an arbitrary stimulus variable and may in principle be high-dimensional. In the synthetic demonstrations; however, a deliberately restricted two-dimensional stimulus space was used,

$$S_{\mathrm{syn}}=\left\{s_{1},s_{2},\ldots ,s_{n}\right\},\;s_{i}=\left[\begin{array}{c} x_{i1}\\ x_{i2} \end{array}\right]\in R^{2}.$$

This restriction was methodological rather than theoretical. It allowed the relation among stimulus geometry, target variables, and candidate category maps to be visualised directly while keeping the generative structure fully controlled. The low-dimensional demonstrations were therefore used to isolate the formal logic of target-preserving compression, not to model the full dimensionality of natural perceptual spaces. In this setting, each candidate category map assigned every sampled stimulus point $s_{i}$ to a category label $C_{i}$. The same stimulus set was assigned multiple target variables. In the flat stimulus-space demonstrations, four targets were defined:

T∈{Y,A,V,G},

where *Y* denotes an identity-like target, *A* an action-like target, *V* a nuisance target, and *G* a geometric target. The identity target *Y* indicated the latent cluster or individual-like class from which a stimulus was generated. The action target *A* grouped stimuli according to a shared consequence or response-relevant class. The nuisance target *V* represented a variable that could structure the stimulus set without being relevant to identity or action. The geometric target *G* described a partition based on spatial organisation in the two-dimensional feature space. These targets were not intended as empirical claims about actual visual categories. They served as controlled variables that made it possible to ask how the same category map can preserve different kinds of information. This use of multiple possible targets follows the general idea that categories support different forms of generalisation depending on which structure is treated as relevant [5,9,10,12]. The demonstrations, therefore, separate the existence of a category partition from the question of which target variable that partition preserves. For each synthetic demonstration, stimulus points were sampled from Gaussian clusters with fixed means and covariance structures, with equal numbers of samples drawn per latent cluster. Unless otherwise noted, the same sampled stimulus set was then evaluated under alternative target assignments and category maps. All conclusions in the synthetic demonstrations were based on information-theoretic quantities computed from the sampled category and target variables. Category complexity was quantified by $H(C_{m})$. Target preservation was quantified by $I(C_{m};T)$ and $\mathrm{NMI}\left(C_{m},T\right)=I\left(C_{m};T\right)/H\left(T\right)$. Residual target uncertainty was quantified by $H(T|C_{m})$. In the perturbation analysis, robustness was quantified as the preservation of $\mathrm{NMI}(C_{m},T)$ across perturbation levels σ. Thus, the visual stimulus plots illustrate the controlled construction, whereas the conclusions are drawn from the corresponding quantitative metrics. In the synthetic demonstrations, the same low-dimensional stimulus space was evaluated under several alternative target assignments and category maps. In the flat demonstrations, the sampled stimuli were analysed relative to identity-like, action-like, nuisance, and geometric targets. Candidate category maps were then defined to preserve identity, action, geometric, nuisance, random, or fully collapsed one-category structure. In the perturbation analysis, Gaussian noise was added to the stimulus coordinates before re-evaluating fixed baseline category-assignment rules. In the hierarchical demonstration, a separate stimulus space was constructed such that subordinate, basic-level, and broader action/superordinate-like targets could all be defined over the same points. This arrangement allowed category level to be treated explicitly as a target-dependent compression of the same underlying stimulus space.

#### 2.3.2. Candidate Category Maps

Candidate category maps were defined over the same stimulus space. Each candidate map *m* was treated as a function

$$f_{m}:S_{\mathrm{syn}}\to C_{m},$$

where $C_{m}=f_{m}\left(S_{\mathrm{syn}}\right)$ denotes the category variable induced by map *m*. In the flat demonstrations, the candidate maps were

$$M_{\mathrm{flat}}=\left\{m_{Y},m_{A},m_{G},m_{V},m_{R},m_{0}\right\}.$$

Here, $m_{Y}$ preserved the identity-like target, $m_{A}$ preserved the action-like target, $m_{G}$ followed the geometric structure of the stimulus space, $m_{V}$ preserved the nuisance variable, $m_{R}$ assigned random category labels, and $m_{0}$ assigned all stimuli to a single category. The collapsed map was defined as

$$C_{m_{0},i}=1\;\mathrm{for}\;\mathrm{all}\;i.$$

Thus, each category map produced a category variable $C_{m}$, but the meaning of that category variable depended on the map that generated it. Some maps were aligned with a target variable, whereas others were deliberately misaligned. This distinction is important because category complexity alone does not imply target usefulness. A category map can have several categories and therefore non-zero entropy $H(C_{m})$, while preserving little or no information about the target variable of interest. These maps were evaluated against the defined target variables using category entropy, mutual information, conditional entropy, and normalised mutual information. Category entropy $H(C_{m})$ quantified the complexity of the category labels produced by map *m*. Mutual information $I(C_{m};T)$ quantified how much information the map preserved about a target variable *T*. Conditional entropy $H(T|C_{m})$ quantified how much target uncertainty remained after the category label was known. Normalised mutual information was computed as

$$\mathrm{NMI}\left(C_{m},T\right)=\frac{I(C_{m};T)}{H(T)}.$$

This expressed preserved information as the fraction of target uncertainty removed by the category map. The notation $C_{m}$ is used here to emphasise that the category variable depends on the selected map *m*. This allowed category complexity and target preservation to be separated explicitly [13,14,16,17]. The corresponding notation for the flat synthetic demonstrations is summarised in Table 2.

#### 2.3.3. Demonstrations 1–3: Flat Stimulus Space

Demonstration 1 evaluated whether compression alone was sufficient for category usefulness. Several category maps were compared over the same stimulus space. The key comparison was between category complexity,

$$H(C_{m}),$$

and preserved target information,

$$I\left(C_{m};Y\right)\;\mathrm{and}\;I\left(C_{m};A\right).$$

This demonstration tested whether category maps with similar entropy could differ in target relevance. It also connected the formal analysis to earlier work treating category utility as a relation between category structure and informative features [3,4].

Demonstration 2 evaluated the same category maps against multiple target variables. Each map was compared with identity *Y*, action *A*, nuisance condition *V*, and geometric structure *G*:

$$\mathrm{NMI}\left(C_{m},T\right),\;T\in \left\{Y,A,V,G\right\}.$$

This demonstration tested whether category usefulness was an intrinsic property of a category map or a relation between a map and a specified target variable. In information-theoretic terms, the same category variable $C_{m}$ can have high $\mathrm{NMI}(C_{m},T)$ for one target *T*, but low $\mathrm{NMI}(C_{m},T^{\prime })$ for another target $T^{\prime }$:

$$\mathrm{NMI}\left(C_{m},T\right)\neq \mathrm{NMI}\left(C_{m},T^{\prime }\right).$$

Therefore, usefulness was defined relative to the target that the category system was expected to preserve.

Demonstration 3 examined robustness under sensory perturbation. Noise was added to the stimulus features, producing perturbed stimulus instances

(8) $$s_{i}^{\left(\sigma \right)}=s_{i}+\varepsilon_{i},\;\varepsilon_{i}∼N\left(0,\sigma^{2}I\right),$$

where σ denotes perturbation strength. Fixed baseline classification rules were then re-evaluated across perturbation levels. Robustness was quantified as the stability of normalised mutual information under perturbation:

(9) $$R_{m}\left(T,\sigma \right)=\mathrm{NMI}\left(C_{m,\sigma },T\right).$$

Here, $C_{m,\sigma }$ denotes the category variable induced by map *m* when applied at perturbation strength σ. A robust target-preserving map should retain relatively high $R_{m}\left(T,\sigma \right)$ as the stimulus coordinates become noisier, whereas a non-aligned map should remain uninformative about the target. This demonstration tested whether target-preserving category maps retained relevant information when the stimulus space was degraded.

#### 2.3.4. Demonstration 4: Hierarchical Category Maps

A separate synthetic demonstration introduced a hierarchical stimulus space. Stimuli were organised such that subordinate identity, basic class, and broader action/superordinate-like targets could all be defined over the same points:

$$T_{\mathrm{hier}}\in \left\{Y_{\mathrm{sub}},Y_{\mathrm{basic}},A\right\}.$$

Candidate category maps were constructed at corresponding levels:

$$M_{\mathrm{hier}}=\left\{m_{\mathrm{sub}},m_{\mathrm{basic}},m_{\mathrm{act}},m_{G},m_{R},m_{0}\right\}.$$

Here, $m_{\mathrm{sub}}$ denotes the subordinate map, $m_{\mathrm{basic}}$ the basic-level map, and $m_{\mathrm{act}}$ the action/superordinate-like map. This demonstration tested whether classification level could be interpreted as a target-dependent compression. A subordinate category map preserves fine identity distinctions but has higher category complexity. A basic-level category map discards some subordinate distinctions while preserving broader class structure. An action/superordinate-like map compresses further and preserves still broader target structure. This formulation is closely related to the classical idea that category levels differ in their balance between informativeness and economy [2,6,7]. In the present analysis, however, this balance was expressed directly in terms of

$$H\left(C_{m}\right),\;I\left(C_{m};T\right),\;\mathrm{NMI}\left(C_{m},T\right).$$

Thus, subordinate, basic, and action/superordinate-like classifications were treated as alternative compressions of the same stimulus space, whose usefulness depended on which target variable was to be preserved.

### 2.4. Neural-Network Extension

#### 2.4.1. Image Dataset and Target Variables

To examine whether the same information-theoretic analysis could be applied to learned visual representations, an exploratory neural-network demonstration was carried out using a pretrained ResNet-50 model [33]. ResNet-50 was used here as a fixed feature extractor, not as a model retrained for the present task. CIFAR-10 images [34] were used as a compact natural-image stimulus set. This extension follows the broader use of deep neural networks as models of hierarchical visual representation and as comparison systems for biological object recognition [23,26,27,30]. Each image $s_{i}$ was assigned to four target variables:

$$Y_{\mathrm{obj}},\;Y_{\sup},\;A,\;V.$$

The object-class target $Y_{\mathrm{obj}}$ corresponded to the ten CIFAR-10 categories:

$$Y_{\mathrm{obj}}\in \left\{\mathrm{airplane},\mathrm{automobile},\mathrm{bird},\mathrm{cat},\mathrm{deer},\mathrm{dog},\mathrm{frog},\mathrm{horse},\mathrm{ship},\mathrm{truck}\right\}.$$

These CIFAR-10 labels are treated here as basic-level-like object classes rather than as subordinate categories. The superordinate target $Y_{\sup}$ distinguished animals from vehicles:

$$Y_{\sup}\in \left\{\mathrm{animal},\mathrm{vehicle}\right\}.$$

The environment- or affordance-like target *A* distinguished air-, land-, and water-associated categories:

A∈{air,land,water}.

The nuisance target *V* denoted the image-transformation condition:

V∈{clean,blurred,pixelated,noise-perturbed}.

Here, *V* was treated as a nuisance variable only relative to object-relevant targets. If the target were to detect image degradation, *V* would be target-relevant. In the present analysis; however, *V* was used to test whether unsupervised activation-space partitions preserved transformation condition rather than object-relevant category structure. The nuisance label was therefore used in two complementary ways. First, it was treated as a target in its own right, allowing the analysis to test whether unsupervised layer-derived category maps preserved image condition. Second, it was used as a conditioning variable, allowing object information to be measured within nuisance conditions. This conditional analysis directly tested whether object-class information remained detectable after the nuisance structure of the image set was taken into account. Here, $y_{i}$ denotes the original CIFAR-10 label of image $s_{i}$. The mapping from CIFAR-10 labels to the superordinate target was defined as follows:

$$Y_{\sup}\left(s_{i}\right)=\left\{\begin{array}{cc} \mathrm{animal}, & \mathrm{if}\;y_{i}\in \left\{\mathrm{bird},\mathrm{cat},\mathrm{deer},\mathrm{dog},\mathrm{frog},\mathrm{horse}\right\},\\ \mathrm{vehicle}, & \mathrm{if}\;y_{i}\in \left\{\mathrm{airplane},\mathrm{automobile},\mathrm{ship},\mathrm{truck}\right\}. \end{array}\right.$$

The environment- or affordance-like target was defined as

$$A\left(s_{i}\right)=\left\{\begin{array}{cc} \mathrm{air}, & \mathrm{if}\;y_{i}\in \left\{\mathrm{airplane},\mathrm{bird}\right\},\\ \mathrm{water}, & \mathrm{if}\;y_{i}\in \left\{\mathrm{ship}\right\},\\ \mathrm{land}, & \mathrm{if}\;y_{i}\in \left\{\mathrm{automobile},\mathrm{cat},\mathrm{deer},\mathrm{dog},\mathrm{frog},\mathrm{horse},\mathrm{truck}\right\}. \end{array}\right.$$

This target was not treated as a literal action label. It was included as a constructed coarse consequence-relevant grouping, allowing the same layer-derived category maps to be evaluated against a target that was broader than the CIFAR-10 object-class target but different from the animal–vehicle distinction.

#### 2.4.2. Clean-Only and Pooled Nuisance Scenarios

Two ResNet-50 scenarios were analysed. In the clean-only object run, 300 images from each CIFAR-10 class were selected, producing 10×300=3000 clean images. This scenario tested whether unsupervised activation-space partitions preserved object-relevant information when systematic nuisance variation was absent. In the clean-only run, *V* was degenerate because all images belonged to the clean condition. Nuisance information was therefore treated as zero by convention and was used only as a reference baseline, not as a meaningful normalised target. In the pooled nuisance run, the same number of original images was selected, but each image was represented in four versions: clean, blurred, pixelated, and noise-perturbed. This produced 10×300×4=12,000 exported images, from which 3000 images were selected for the activation analysis. The activation analysis was limited to 3000 images for computational comparability with the clean-only run. This subset was sampled after image generation and was stratified by object class $Y_{\mathrm{obj}}$ where possible. This scenario tested whether the same unsupervised procedure preserved object-relevant information or instead captured transformation-induced nuisance structure when image condition varied systematically. The contrast between the two scenarios was therefore designed to distinguish object-relevant preservation from nuisance-condition preservation.

#### 2.4.3. Network Model and Layer Activations

For each selected ResNet-50 layer *l*, an activation vector was extracted for each image:

(10) $$z_{i,l}=\phi_{l}\left(s_{i}\right),$$

where $s_{i}$ denotes image *i*, $\phi_{l}\left(\cdot \right)$ denotes the transformation implemented by ResNet-50 up to layer *l*, and $z_{i,l}$ denotes the corresponding activation vector. Six layers were selected to sample early, intermediate, late, and final network representations:

$$\begin{array}{cc} \; & \mathrm{activation}\_1\_\mathrm{relu},\;\mathrm{activation}\_10\_\mathrm{relu},\;\mathrm{activation}\_22\_\mathrm{relu},\\ \; & \mathrm{activation}\_40\_\mathrm{relu},\;\mathrm{activation}\_49\_\mathrm{relu},\;\mathrm{avg}\_\mathrm{pool}. \end{array}$$

These layers were selected to sample the representational hierarchy from early convolutional processing to the final pooled representation before classification. The layer-derived activation spaces were treated as learned representational spaces in which object category, broader category structure, affordance-like structure, and nuisance condition could be preserved to different degrees [26,29,32]. Before clustering, activation vectors were standardised and reduced by principal component analysis. Thirty principal components were retained, yielding a reduced feature vector

$${\tilde{z}}_{i,l}\in R^{30}$$

for each image and layer. For each scenario, layer, and value of *k*, clustering was applied to the standardised PCA-reduced activation matrix. *k*-means was run with five replicates and a maximum of 200 iterations per replicate. The random seed was fixed at 1 to make image selection and clustering reproducible. For each layer *l* and number of clusters *k*, this produced a layer-specific category map

(11) $$g_{l,k}:{\tilde{z}}_{i,l}\to C_{l,k},$$

where $C_{l,k}$ denotes the cluster label assigned to image *i*. Thus, $C_{l,k}$ was treated as the category variable induced by unsupervised clustering of the activation geometry at layer *l*.

For the conditional network controls, the clustering procedure was repeated across multiple *k*-means initialisations across a more densely sampled ResNet-50 layer hierarchy. These controls used 17 sampled layers spanning the ResNet-50 hierarchy, from activation_4_relu through activation_49_relu, together with avg_pool. Intermediate activation layers were sampled in steps of three residual activation blocks. The controls used k=10 and 30 principal components, matching the ten CIFAR-10 object classes while retaining the dimensionality used in the original network analysis. For each analysed layer, *k*-means clustering was repeated across 20 random initialisations. Two nuisance conditions were analysed. The first used the strong pooled-nuisance image set, in which object identity was crossed with visually salient transformation conditions. The second used a milder nuisance image set matched conceptually to the supervised-readout analyses. Together, these controls tested whether unsupervised layer-derived category maps preserved object information, nuisance information, or object information that remained detectable after conditioning on nuisance condition.

#### 2.4.4. Information-Theoretic Evaluation of Layer-Derived Category Maps

For each analysed layer *l* and cluster number *k*, the entropy of the induced category variable $C_{l,k}$ was computed:

(12) $$H\left(C_{l,k}\right)=-\sum_{c}p\left(c\right)\log_{2}p\left(c\right).$$

This value quantified the complexity of the unsupervised layer-derived category map. The same category map was then evaluated against each target variable:

$$T\in \{Y_{\mathrm{obj}},Y_{\sup},A,V\}.$$

Preserved target information was quantified by

$$I\left(C_{l,k};Y_{\mathrm{obj}}\right),\;I\left(C_{l,k};Y_{\sup}\right),\;I\left(C_{l,k};A\right),\;I\left(C_{l,k};V\right).$$

The corresponding normalised values were computed as

(13) $$\mathrm{NMI}\left(C_{l,k},T\right)=\frac{I(C_{l,k};T)}{H(T)}.$$

Thus, $\mathrm{NMI}(C_{l,k},T)$ measured the fraction of uncertainty about target *T* that was removed by knowing the layer-derived cluster label $C_{l,k}$. A value close to zero indicated that the cluster labels preserved little information about the target. A value close to one indicated that the cluster labels almost fully determined the target. This analysis quantified whether a layer-derived category map preserved object-class information, superordinate animal–vehicle information, environment- or affordance-like information, or nuisance-condition information. Importantly, the procedure did not train a supervised classifier for each target. Instead, it asked which target variables were already preserved by unsupervised partitions of the activation space. This distinction was central to the neural-network extension: a layer-derived category map could be structured and non-trivial but still preserve information primarily about the nuisance condition rather than the intended object-relevant target.

To determine whether object information remained after accounting for nuisance condition, conditional object information was additionally computed as

(14) $$\mathrm{NMI}\left(C_{l,k},Y_{\mathrm{obj}}|V\right)=\frac{I(C_{l,k};Y_{\mathrm{obj}}|V)}{H(Y_{\mathrm{obj}}|V)}.$$

A within-nuisance permutation baseline was used for this conditional measure. Object labels were shuffled separately within each nuisance condition, and conditional normalised information was recomputed for each permutation. The null-corrected conditional object value was obtained by subtracting the mean within-nuisance permutation baseline from the observed conditional value. This analysis asked whether object information was retained within nuisance conditions, rather than merely co-varying with nuisance structure. For each layer-derived category map, object information, nuisance information, conditional object information given nuisance condition, and null-corrected conditional object information were computed. The analysis therefore separated three possibilities: object identity could dominate the unsupervised category geometry, nuisance condition could dominate the geometry, or object information could remain detectable within nuisance conditions even when nuisance condition dominated the overall geometry.

### 2.5. Implementation and Reproducibility

All simulations and neural-network analyses were implemented in MATLAB R2024b. Synthetic demonstrations generated the stimulus spaces, target variables, candidate category maps, information-theoretic quantities, and figures directly from the analysis scripts. The neural-network extension used CIFAR-10 image batches [34], a pretrained ResNet-50 model [33], principal component analysis, *k*-means clustering [35], and the same entropy and mutual-information functions used for the synthetic demonstrations [13,14]. Because the synthetic demonstrations used known generative structures and explicitly defined category maps, the main comparisons were information-theoretic rather than inferential. The reported values quantify the preservation properties of each category map directly, rather than estimating population effects from sampled participants or empirical trials. The neural-network extension was treated as an exploratory computational demonstration of the same principle in learned representations.

## 3. Results

The analyses below were organised around a single quantitative contrast. If compression alone were sufficient for evaluating categorisation, then the usefulness of a category map should be largely determined by its category entropy, H(C). Under the target-preservation account; however, category entropy and usefulness can dissociate: two category maps can have similar complexity while preserving very different amounts of information about a target variable *T*. The decisive quantities are therefore not only H(C), but also I(C;T), H(T∣C), and the normalised preservation score I(C;T)/H(T). Each demonstration tests one aspect of this dissociation. Demonstration 1 compares category complexity with preserved identity and action information. Demonstration 2 tests whether the same category map can be useful for one target but not another. Demonstration 3 examines whether preserved information remains stable under perturbation. Demonstration 4 applies the same logic to hierarchical category levels. Demonstration 5 extends the analysis to layer-derived category maps in a pretrained neural network.

### 3.1. Demonstration 1: Compression Alone Is Insufficient

**Figure 1:**
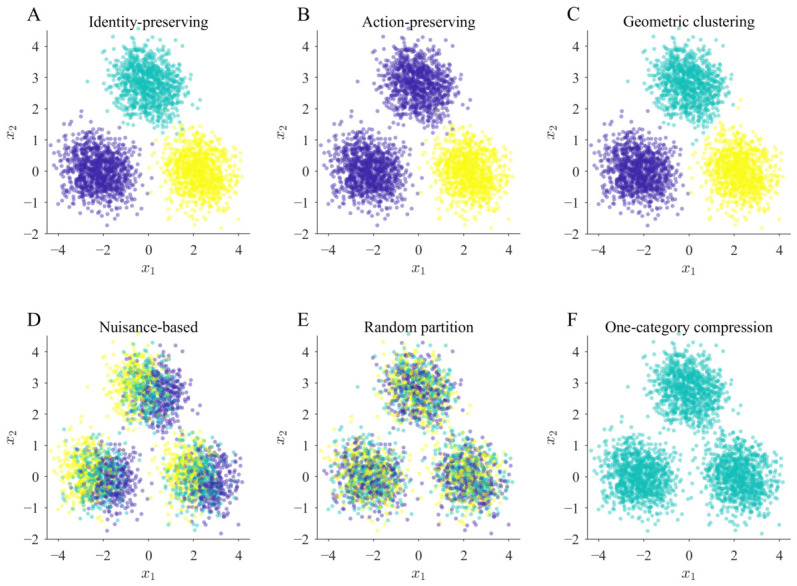
Candidate category maps over the same two-dimensional stimulus space. Panels show (**A**) identity-preserving, (**B**) action-preserving, (**C**) geometric clustering, (**D**) nuisance-based, (**E**) random partition, and (**F**) one-category compression maps. The stimulus coordinates are identical across panels; only the category assignment changes. The figure illustrates that the same stimulus geometry can support several possible compressions, only some of which preserve the target variable of interest. Colours denote category assignments within each panel. The colour coding is panel-specific and does not imply correspondence across different category maps.

**Figure 2:**
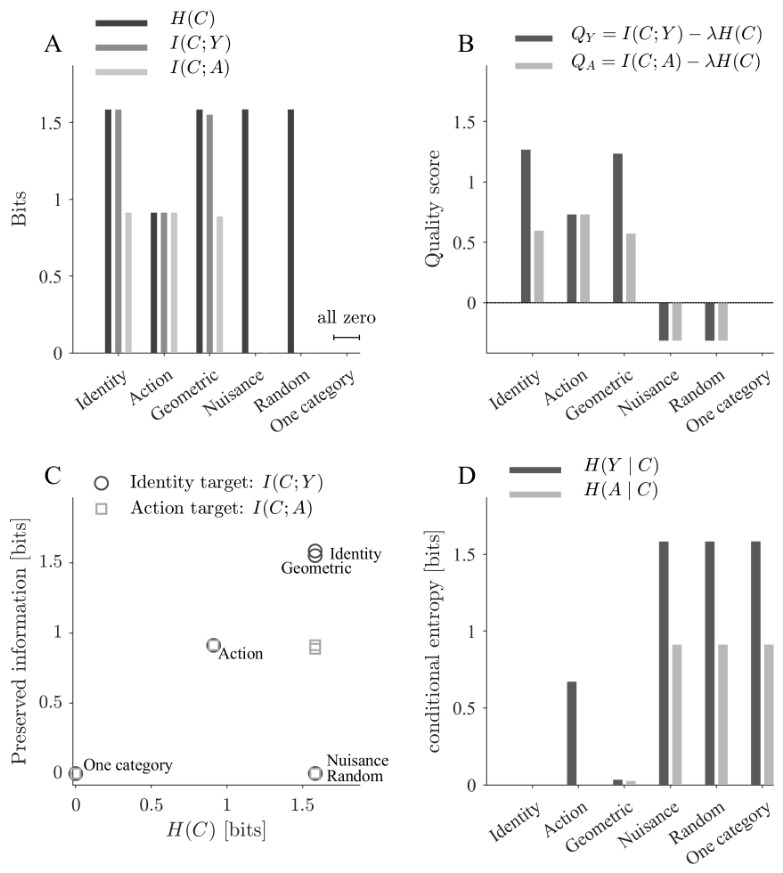
Information-theoretic comparison of the candidate category maps. (**A**) Category entropy H(C), identity information I(C;Y), and action information I(C;A). (**B**) Target-specific quality scores $Q_{Y}=I\left(C;Y\right)-\lambda H\left(C\right)$ and $Q_{A}=I\left(C;A\right)-\lambda H\left(C\right)$. (**C**) Preserved target information plotted against category entropy. Circles indicate the identity target I(C;Y) and squares indicate the action target I(C;A); text labels indicate the candidate category maps. (**D**) Conditional entropy after categorisation, showing the remaining identity uncertainty H(Y∣C) and action uncertainty H(A∣C). Together, the panels show that category complexity alone is insufficient: useful compression requires preservation of the selected target variable.

The first demonstration evaluated six candidate category maps over the same two-dimensional stimulus space (Figure 1). The stimulus coordinates were held constant across panels. Only the assignment of points to categories was changed. The identity-preserving, action-preserving, and geometric maps imposed partitions that were aligned with different target structures, whereas the nuisance-based and random maps imposed non-trivial partitions that were not aligned with the identity or action targets. The one-category map represented maximal compression by assigning all stimuli to a single class. The visual comparison in Figure 1 shows why the geometry of the stimulus space is not sufficient to define a category map. The same three-cluster distribution can be partitioned as an identity map, an action map, a geometric map, a nuisance map, a random map, or a collapsed one-category map. These alternatives differ not because the stimuli differ, but because different variables are preserved by the mapping from stimuli to categories. The information-theoretic summaries in Figure 2A–D provide the quantitative test of this claim. The relevant comparison was between category entropy, H(C), and the information that the category variable preserved about each target, I(C;Y) and I(C;A), consistent with the distinction between category complexity and preserved target information [3,4,13,14]. If compression alone were sufficient, maps with non-zero category entropy should be useful because they impose a structured reduction of the stimulus space. If target preservation is required, however, category entropy and usefulness should dissociate. This dissociation was observed. In Figure 2A, the identity-preserving map had high category entropy and preserved the identity target almost fully, whereas the action-preserving map had lower category entropy but preserved the action target. By contrast, the nuisance-based and random maps retained category complexity but preserved little or no information about the identity or action targets. The one-category map provided maximal compression but left the target variables largely unresolved. Thus, the decisive quantity was not compression alone, but the amount of target information retained by the compressed category variable. The quality-score and conditional-entropy panels express the same dissociation in two complementary ways. In Figure 2B, penalising category complexity favoured category maps that preserved the selected target rather than merely producing many categories. Figure 2C shows that preserved target information did not follow category entropy alone: maps with similar entropy differed in the information they retained about identity or action. In Figure 2D, conditional entropy showed the residual uncertainty after categorisation: target-aligned maps reduced uncertainty about the corresponding target, whereas nuisance-based, random, and one-category maps left substantial identity or action uncertainty. Therefore, compression was not sufficient. Relevant information had to be preserved by the compression.

### 3.2. Demonstration 2: Preserved Information Depends on the Target

**Figure 3:**
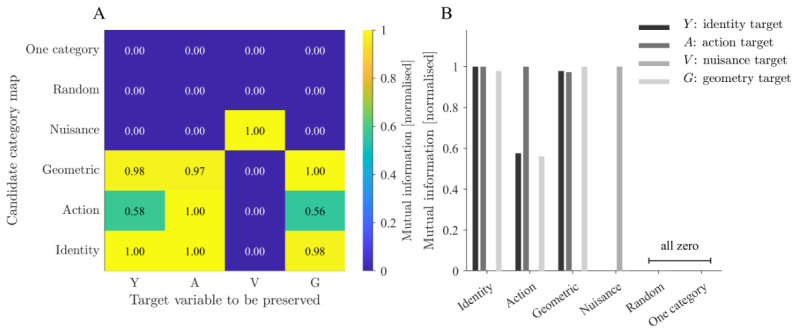
Target-specific preservation by the same candidate category maps. (**A**) Normalised mutual information between each candidate category map and each target variable. Rows correspond to category maps and columns correspond to target variables: identity *Y*, action *A*, nuisance condition *V*, and geometric structure *G*. (**B**) The same values shown as grouped bars. The identity, action, geometric, and nuisance maps show high normalised mutual information for different targets, whereas the random and one-category maps preserve no target information in this construction.

The second demonstration evaluated the same candidate category maps against four target variables: identity *Y*, action *A*, nuisance condition *V*, and geometric structure *G* (Figure 3A,B). For each map–target pair, usefulness was quantified as normalised mutual information, I(C;T)/H(T), where *T* denotes the target under evaluation. Figure 3A shows the resulting target-by-map matrix of normalised mutual information, whereas Figure 3B presents the same values as grouped bars. The result showed that no category map was intrinsically useful in a target-independent sense. Rather, usefulness depended on the measured relation between the category map and the variable that was to be preserved, consistent with target-dependent accounts of compression and category utility [3,4,16,17]. The identity-preserving map preserved the identity target fully and, because of the construction of the stimulus space, also preserved the action target. It additionally preserved most of the geometric target. The action-preserving map fully preserved the action target but preserved only part of the identity and geometric targets. The geometric map preserved the geometric target and also preserved much of the identity and action structure because the geometric clusters were aligned with the underlying stimulus clusters. The nuisance map preserved the nuisance target but preserved essentially none of the identity, action, or geometric targets. The random and one-category maps preserved no target information in this construction. Together, Figure 3A, B make the target dependence of category usefulness explicit in quantitative form. A nuisance category is useful if nuisance condition is the target but useless if object identity, action relevance, or geometric structure is the target. Likewise, a coarse action category can be useful for action selection while being insufficient for subordinate identity recognition. The same category map can therefore receive a high value of I(C;T)/H(T) for one target and a low value for another. Category maps therefore require an explicit target variable for evaluation; the relevant question is not whether a map compresses the stimulus space, but which variable remains informative after compression.

### 3.3. Demonstration 3: Robustness of Preserved Information Under Perturbation

**Figure 4:**
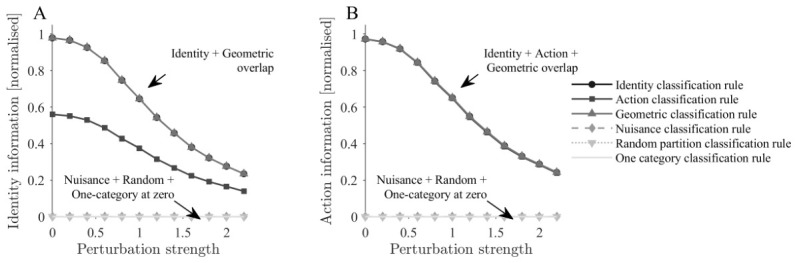
Robustness of preserved target information under sensory perturbation. (**A**) Normalised identity information as a function of perturbation strength. Identity and geometric classification rules overlap closely, whereas nuisance, random, and one-category classification rules overlap at zero. (**B**) Normalised action information as a function of perturbation strength. Identity, action, and geometric classification rules overlap closely because all three preserve the action-relevant structure in the present stimulus construction. Nuisance, random, and one-category classification rules again remain at zero.

The third demonstration examined how preserved information changed under sensory perturbation, separately for identity-relevant information measured as I(C;Y)/H(Y) (Figure 4A) and action-relevant information measured as I(C;A)/H(A) (Figure 4B). Perturbations were introduced into the stimulus features, and fixed baseline classification rules were applied to the perturbed stimulus coordinates. The analysis examined whether category-assignment rules aligned with the target continued to preserve identity-relevant and action-relevant information as sensory noise increased. This question is related to the broader problem of whether representations preserve task-relevant structure while becoming invariant, or at least robust, to nuisance variation [25,32]. For identity-relevant information the identity and geometric classification rules produced nearly overlapping robustness profiles (Figure 4A). Both preserved high-identity information under weak perturbation, followed by a gradual decline as perturbation strength increased. The action classification rule preserved less identity information, but remained above zero across the tested perturbation range because the action partition retained part of the identity structure. Nuisance, random, and one-category classification rules overlapped at zero, indicating that these classification rules preserved essentially no identity-relevant information. For action-relevant information, the identity, action, and geometric classification rules overlapped closely (Figure 4B). This is expected from the construction of the stimulus space: the identity and geometric distinctions retained the action-relevant grouping, and the action classification rule preserved it directly. As perturbation increased, preserved action information decreased gradually but remained substantially above the nuisance, random, and one-category classification rules. Thus, robustness depended on alignment between the classification rule and the target variable, not merely on the existence of a category partition. Representative normalised mutual information values at selected perturbation strengths are reported in Table 3.

### 3.4. Demonstration 4: Hierarchical Category Maps over the Same Stimulus Space

**Figure 5:**
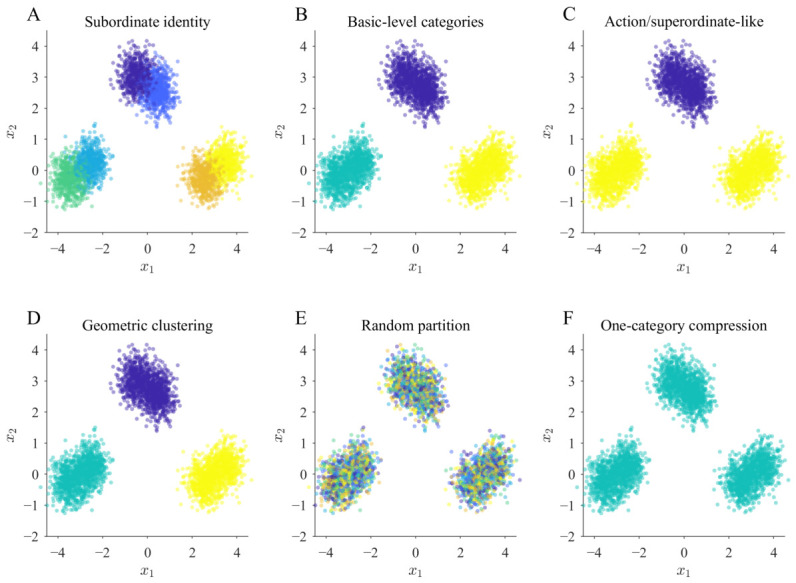
Hierarchical category maps over the same stimulus space. Panels show (**A**) subordinate identity, (**B**) basic-level category, (**C**) action/superordinate-like, (**D**) geometric clustering, (**E**) random partition, and (**F**) one-category compression maps in the hierarchical demonstration. The panels illustrate that subordinate, basic-level, action/superordinate-like, geometric, random, and collapsed maps impose different compressions on the same underlying stimulus distribution. Colours denote category assignments within each panel. The colour coding is panel-specific and does not imply correspondence across category maps.

**Figure 6:**
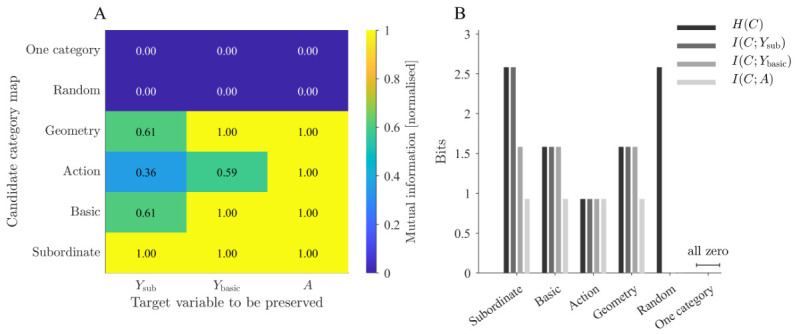
Information-theoretic analysis of hierarchical category maps. (**A**) Normalised mutual information between each hierarchical category map and each target variable. Rows correspond to candidate maps and columns correspond to target variables. (**B**) Category entropy and preserved target information for subordinate identity $Y_{\mathrm{sub}}$, basic-level target $Y_{\mathrm{basic}}$, and action target *A*. The subordinate map preserves the most fine-grained target at higher complexity, whereas basic and action maps impose stronger compression while preserving broader target variables. The random map retains category entropy without useful target preservation, and the one-category map is an all-zero case.

The fourth demonstration examined hierarchical category maps over a stimulus space in which subordinate identities, basic-level categories, and a broader action/superordinate-like target were defined over the same points (Figure 5A–F). The demonstration specifically asked whether category level can be interpreted as target-dependent compression. Candidate maps were constructed at corresponding levels of specificity, together with geometric, random, and one-category alternatives. This allowed category level to be treated as an information-preservation problem rather than as a fixed naming hierarchy, with each level evaluated by the information it preserved about subordinate identity, basic-level structure, and action-relevant grouping. Figure 5 is used as a construction figure: it shows how subordinate, basic-level, action/superordinate-like, geometric, random, and collapsed maps assign category labels to the same points. The quantitative evaluation is shown in Figure 6, where each map is evaluated against subordinate identity, basic-level, and action-relevant targets using normalised mutual information and category entropy. This framing is related to classical work on basic-level categories, in which category levels are understood as differing in their balance between informativeness, generality, and economy [2,6,7].

The visual partitions in Figure 5A–F show that the same hierarchical stimulus space can be compressed at different levels. The subordinate map preserved the finest identity distinctions. The basic map merged subordinate identities into broader kinds. The action-level map compressed the space further by preserving the action-relevant grouping. The geometric map recovered much of the basic/action structure because spatial organisation was aligned with these broader targets. The close correspondence between the basic-level map and the geometric clustering map is informative: in this synthetic construction, the basic-level categories coincide with the dominant geometry of the stimulus space. Thus, the geometric map preserves basic-level information not because it was defined by the basic-level labels, but because the basic-level labels are aligned with compact regions of the feature space. The random map retained category complexity without target alignment, whereas the one-category map collapsed all stimuli into a single class. This should not be interpreted as a general claim that basic-level categories are always geometrically obvious; rather, it shows that category level and stimulus-space geometry can coincide when the relevant features are organised in clustered form.

In Figure 6A, the subordinate map preserved subordinate information fully, whereas the basic, action, and geometric maps preserved different broader targets as quantified by target-specific normalised mutual information. In Figure 6B, the subordinate map preserved the finest target at higher category entropy, the basic map discarded subordinate detail while preserving basic and action-relevant information, and the action map imposed the strongest non-trivial target-aligned compression while preserving the action target. The geometric map preserved basic and action information because of the spatial structure of the synthetic stimulus space. Random and one-category maps were not useful for the defined targets: the random map retained entropy without preserving target information, and the one-category map collapsed both complexity and target preservation. Thus, the quantitative result is not simply that some partitions are more detailed than others. Rather, category level is useful only relative to the target variable that must be preserved.

This result shows that category level can be interpreted as a quantitative trade-off between complexity and preserved target information. Subordinate, basic, and action/superordinate-like categories are not merely points on a naming hierarchy. They are different compressions of the same stimulus space, each preserving a different target structure. In this sense, category level is target-dependent rather than category-level independent. The same partition may be appropriate for one inferential or behavioural demand and inappropriate for another. This interpretation is consistent with accounts in which category usefulness depends on the information that categories preserve about relevant properties, rather than on category granularity alone [3,4]. Representative quantitative values from the non-perturbation synthetic demonstrations are summarised in Table 4.

### 3.5. Demonstration 5: Layer-Derived Category Maps in a Pretrained Visual Network

**Figure 7:**
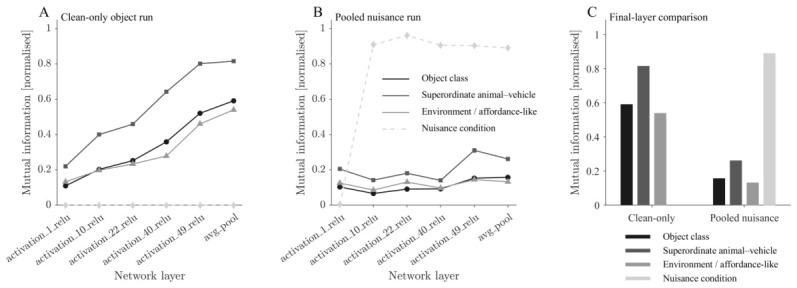
Layer-derived category maps in a pretrained ResNet-50 model. (**A**) Clean-only object run with k=10: normalised mutual information between the layer-derived category map $C_{l,10}$ and each target variable across the analysed network layers. Object-relevant targets, especially the superordinate animal–vehicle target, increased across the network hierarchy, whereas nuisance information was absent. The colours and line styles used in panel A correspond to the legend shown in panel B, which applies to both panels. (**B**) Pooled nuisance run with clean, blurred, pixelated, and noise-perturbed images: nuisance condition became the dominant preserved target, while object-class, superordinate animal–vehicle, and environment- or affordance-like information remained comparatively weak. The line legend shown in panel B applies to both panels A and B. (**C**) Final-layer comparison at avg_pool, showing that clean-only partitions preserved object-relevant information, whereas pooled-nuisance partitions preferentially preserved nuisance condition. Panel C uses the bar legend shown below the axis. All values are normalised mutual information values, $I\left(C_{l,10};T\right)/H\left(T\right)$.

**Figure 8:**
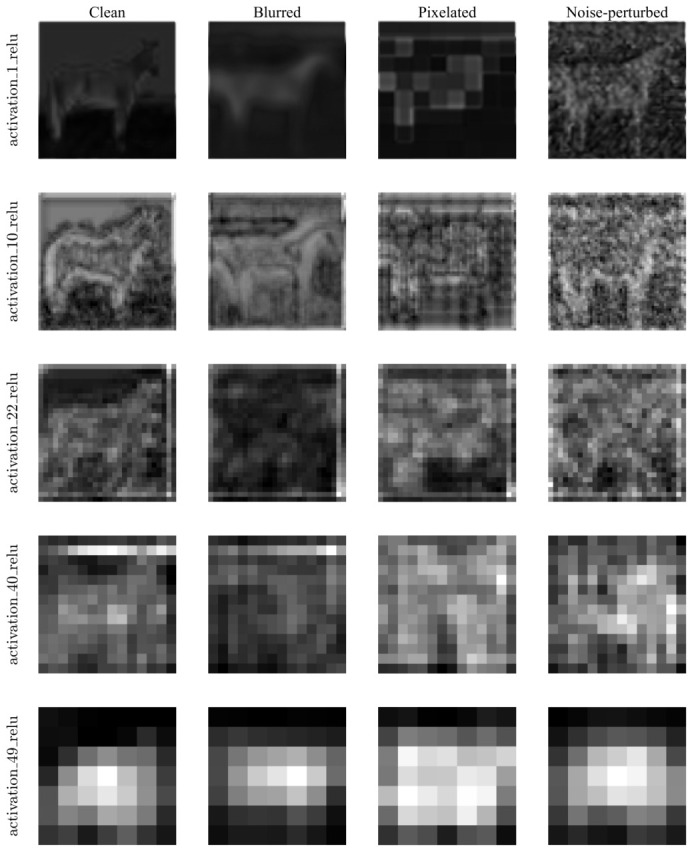
Qualitative example of layer-wise activation structure for one image under different nuisance transformations. Columns show the clean, blurred, pixelated, and noise-perturbed versions of the same input image. Rows show a subset of the analysed ResNet-50 layers: activation_1_relu, activation_10_relu, activation_22_relu, activation_40_relu, and activation_49_relu. The figure illustrates how distortion-dependent structure remains visible across the representational hierarchy and complements the information-theoretic summaries in Figure 7.

The preceding demonstrations used explicitly constructed category maps over controlled stimulus spaces. The fifth demonstration examined whether the same target-preservation logic could be applied to category maps induced from the activation geometry of a pretrained visual network. ResNet-50 activations were extracted for CIFAR-10 images [33,34], and unsupervised *k*-means partitions of the reduced activation spaces were treated as layer-derived category maps. This follows the broader use of deep neural networks as hierarchical visual representation models and as comparison systems for biological object recognition [26,27,30]. These maps were then evaluated quantitatively, using normalised mutual information $I\left(C_{l,k};T\right)/H\left(T\right)$, against object class, superordinate animal–vehicle class, environment- or affordance-like class, and nuisance condition. The main network summary is shown for k=10, because this value matches the number of CIFAR-10 object classes and provides the strongest test of whether unsupervised activation-space partitions preserve object-relevant information rather than information about image-transformation condition when evaluated by $I\left(C_{l,10};T\right)/H\left(T\right)$ (Figure 7A–C). Figure 8 provides a qualitative single-example illustration of how the same object, under different nuisance transformations, is represented across the analysed network layers. This visualisation complements the information-theoretic summaries in Figure 7 by showing that transformation-dependent structure remains visible across much of the representational hierarchy.

In the clean-only object run, object-relevant information increased across the network hierarchy (Figure 7A). Object-class information increased from approximately 0.11 in the first analysed layer to approximately 0.59 at avg_pool. Superordinate animal–vehicle information increased more strongly, from approximately 0.22 to approximately 0.82. The environment- or affordance-like target also increased, from approximately 0.13 to approximately 0.54. Because no image-condition variation was present in this run, nuisance information was treated as zero by convention. Thus, when the stimulus set contained only clean images, unsupervised activation-space partitions increasingly preserved object-relevant information across the network hierarchy. This pattern is consistent with the general view that later layers in deep visual networks tend to support more abstract object-relevant representations [26,29]. In the pooled nuisance run, the pattern changed qualitatively (Figure 7B). The image set contained clean, blurred, pixelated, and noise-perturbed versions of the CIFAR-10 stimuli. Under these conditions, nuisance condition became the dominant preserved target after the first analysed layer. Nuisance information rose to approximately 0.91 at activation_10_relu, reached approximately 0.96 at activation_22_relu, and remained high through avg_pool at approximately 0.89. In contrast, object-class, superordinate animal–vehicle, and environment- or affordance-like information remained much lower throughout the same run. The layer-derived category map was therefore highly informative, but primarily about the nuisance variable rather than the semantic object targets. This result is compatible with the broader concern that activation-space structure can be strongly shaped by image statistics, transformations, or nuisance variation rather than by semantic object category alone [25,32]. The final-layer comparison makes the contrast between scenarios explicit (Figure 7C). At avg_pool, the clean-only run preserved substantial object-relevant information, especially for the superordinate animal–vehicle target. In the pooled nuisance run, object-relevant information was much weaker, whereas nuisance condition was strongly preserved. The same network, same clustering logic, and same information-theoretic evaluation therefore produced different category-map relevance depending on the structure of the stimulus set. When nuisance transformations were absent, object-relevant structure was recovered. When nuisance transformations were pooled with object variation, the unsupervised category map preferentially captured transformation-induced nuisance structure. These values are reported as normalised mutual information, $I\left(C_{l,10};T\right)/H\left(T\right)$, so they quantify the fraction of uncertainty about each target removed by the layer-derived category map. The key quantitative contrast is therefore not simply that the pooled-nuisance run produced a structured partition, but that the dominant preserved target shifted from object-relevant targets in the clean-only run to nuisance condition in the pooled run. Exact values for the k=10 pooled-nuisance run are reported in Table 5.

The pooled-nuisance result raised a critical control question: whether the observed category maps should be interpreted as object-relevant category maps, or whether they mainly reflected image-condition structure. This was addressed by a conditional control analysis. For each layer-derived category map, object information, nuisance information, conditional object information given nuisance condition, and null-corrected conditional object information were computed. The analysis therefore separated three possibilities: object identity could dominate the unsupervised category geometry, nuisance condition could dominate the geometry, or object information could remain detectable within nuisance conditions even when nuisance condition dominated the overall geometry.

**Figure 9:**
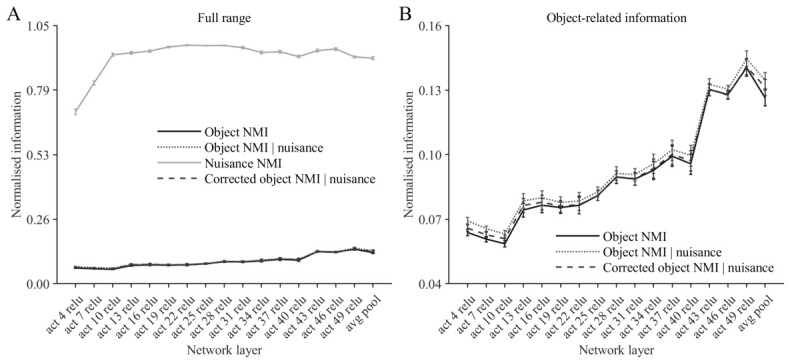
Conditional network control under strong pooled-nuisance variation. Layer-derived category maps were obtained by unsupervised *k*-means clustering of ResNet-50 activations using k=10 and 30 principal components. (**A**) Full-range view of normalised information between the induced category map and object class, nuisance condition, object class conditioned on nuisance condition, and null-corrected conditional object information. (**B**) Zoomed view of the object-related quantities. Under strong nuisance variation, nuisance condition dominated the unsupervised category geometry across the sampled layer hierarchy, whereas object information remained detectable but comparatively weak. Conditional object information closely tracked raw object information, and the null-corrected conditional object signal remained above zero. Lines connect discrete layer-wise estimates for visual guidance only. Error bars denote bootstrap 95% confidence intervals across *k*-means initialisations.

**Figure 10:**
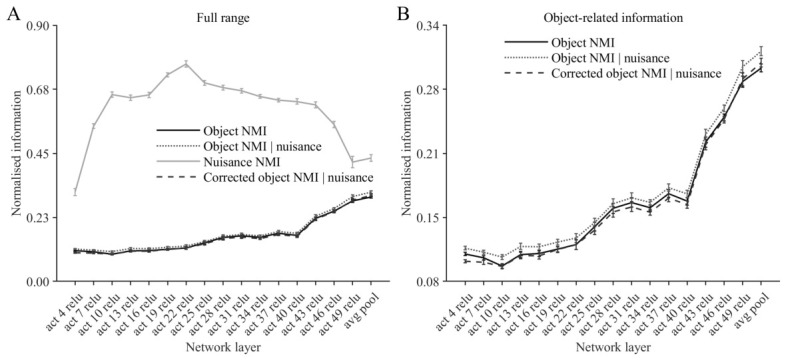
Conditional network control under mild nuisance variation. The same unsupervised *k*-means control was applied to a mild-nuisance image set, using k=10, 30 principal components, and the same sampled ResNet-50 layer hierarchy. (**A**) Full-range view of normalised information between the induced category map and object class, nuisance condition, object class conditioned on nuisance condition, and null-corrected conditional object information. (**B**) Zoomed view of the object-related quantities. Compared with the strong pooled-nuisance condition, nuisance information remained prominent but was less overwhelming, while object-related information increased across later layers. This indicates that nuisance dominance in the strong pooled-nuisance analysis reflected the strength of the imposed transformation structure rather than an absence of object information in the network. Lines connect discrete layer-wise estimates for visual guidance only. Error bars denote bootstrap 95% confidence intervals across *k*-means initialisations.

**Figure 11:**
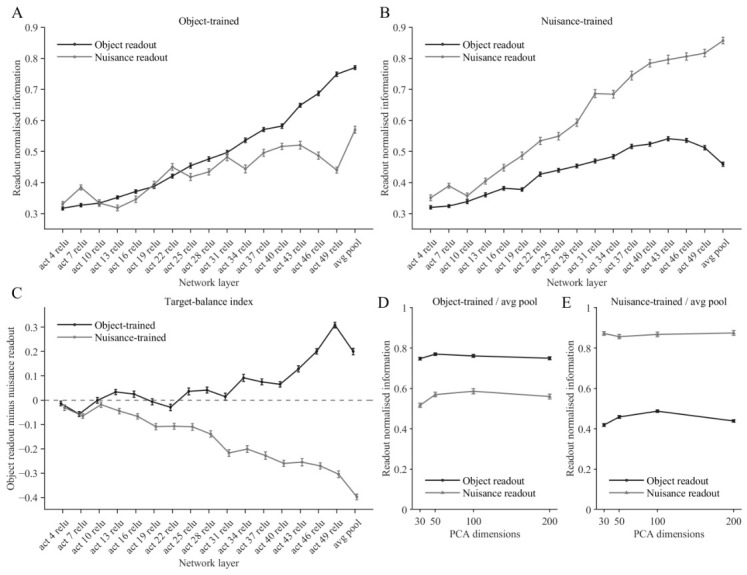
Target-specific supervised readout under controlled mild nuisance variation. (**A**) In object-trained networks, object readout increased across layers and exceeded nuisance readout in later representations. (**B**) In nuisance-trained networks, nuisance readout increased across layers and exceeded object readout. (**C**) The target-balance index, defined as object readout minus nuisance readout, diverged according to the training target. (**D**,**E**) The avg-pool readout pattern was stable across PCA dimensionalities. Points denote means across repeated stratified readout splits; error bars denote bootstrap 95% confidence intervals. Lines connect discrete layer-wise estimates for visual guidance only. Black denotes object readout or object-trained models; grey denotes nuisance readout or nuisance-trained models.

The conditional network controls showed that the variable preserved by unsupervised layer-derived category maps depended strongly on nuisance strength. Under the strong pooled-nuisance condition, nuisance information dominated the induced category maps across the sampled ResNet-50 hierarchy (Figure 9A). Object information was detectable but comparatively weak. The zoomed object-information panel showed that raw object information, conditional object information given nuisance condition, and null-corrected conditional object information followed closely related layer-wise profiles (Figure 9B). Thus, object information was not absent, and it was not merely an artefact of nuisance co-variation, but it was not the dominant structure recovered by unsupervised clustering. A different balance was observed under mild nuisance variation. Nuisance information remained prominent, but its dominance was reduced relative to the strong pooled-nuisance condition (Figure 10A). At the same time, object-related information increased substantially across later layers, reaching markedly higher values than in the strong nuisance control (Figure 10B). Conditional object information and its null-corrected estimate closely tracked the raw object-information profile, indicating that object information remained available within nuisance conditions. These results show that nuisance dominance was graded rather than categorical: unsupervised category maps preferentially recovered nuisance structure when nuisance variation was strong, but object-related structure became increasingly visible when nuisance variation was milder. This neural-network extension provides an important qualification to the synthetic demonstrations. ResNet-50 clearly contains object-relevant information, as shown by the clean-only run. However, when category maps are derived by unsupervised clustering of activation geometry in a stimulus set with strong nuisance transformations, the dominant preserved variable need not be semantic object class. The analysis therefore separates three issues that are often conflated: a representation can be high-dimensional or structured, a category map can have substantial entropy, and the information preserved by that map can still be irrelevant to the intended semantic target. This distinction is central to information-theoretic approaches in which compression is evaluated by the information retained about a specified target variable, not by compression or separability alone [16,17]. A supervised-readout analysis was therefore used as a complementary diagnostic. The unsupervised clustering analysis asks which variable dominates the spontaneous geometry of the representation when no target is imposed at the readout stage. A supervised readout asks a different question: which target information can be recovered by a decoder trained for a specified target. These two analyses are not interchangeable. The first diagnoses dominant representational geometry; the second diagnoses target-specific availability. The supervised-readout analysis was therefore used to test whether target-specific training could shift the balance between object and nuisance information when nuisance variation was controlled. The controlled mild-nuisance readout analysis showed that the balance of preserved information depended on the training target. In object-trained networks, object information increased across later layers and exceeded nuisance information in the final representations (Figure 11A). In nuisance-trained networks, the opposite pattern was observed: nuisance information became the dominant decodable variable (Figure 11B). The target-balance index therefore diverged in opposite directions for object-trained and nuisance-trained networks (Figure 11C). The avg-pool readout pattern was stable across the tested PCA dimensionalities (Figure 11D,E). This result supports the target-relative interpretation of the framework: the same input domain can give rise to category maps whose diagnostic value depends on the target variable that is preserved. The supervised-readout result should be interpreted together with the pooled-nuisance control. Under strong pooled nuisance variation, nuisance condition can dominate unsupervised category geometry. Under controlled mild nuisance variation, target-specific training can shift the dominant decodable variable toward the trained target. These results jointly show that category-map usefulness cannot be inferred from compression or clustering alone. It must be evaluated relative to the target variable and to the strength of competing nuisance structure.

## 4. Discussion

The present analysis supports a simple but important conclusion: categorisation cannot be evaluated by compression alone. A category map reduces the complexity of a stimulus space, but its usefulness depends on what information is preserved by that reduction. Category entropy measures how much categorical structure remains after compression, whereas mutual information with a target variable measures whether that remaining structure is relevant to a specified cognitive, behavioural, or computational target [13,14,16,17]. Across the demonstrations, this conclusion was supported by explicit comparisons between category entropy, H(C), preserved target information, I(C;T), residual target uncertainty, H(T∣C), and normalised mutual information, I(C;T)/H(T). The synthetic demonstrations showed that the same stimulus space can support multiple candidate category maps with sharply different informational consequences. Identity-preserving, action-preserving, geometric, nuisance-based, random, and one-category maps all impose partitions on the same points. Some preserve the target of interest; others do not. The difference cannot be read off from category entropy alone. A random partition can have high entropy and low usefulness. A one-category partition can achieve maximal compression and no usefulness. A nuisance partition can be highly informative, but only about a nuisance target. This result is consistent with earlier accounts in which category utility depends on the relation between category structure and informative properties, rather than on category structure alone [3,4]. This distinction provides an operational link to category-utility and basic-level accounts. Classical work on basic-level categories emphasised that some levels of abstraction are especially useful because they balance informativeness and economy [2,6,7]. The present formulation expresses this idea in information-theoretic terms. A category level is useful when it preserves the target variable required by the task while avoiding unnecessary category complexity. Subordinate, basic, and action/superordinate-like levels therefore need not be treated as fixed cognitive levels with universal value. They can be treated as alternative compressions whose value depends on the target structure that must be retained. The neural-network extension strengthens this conclusion in a learned representational setting. In the clean-only ResNet-50 run, layer-derived category maps increasingly preserved object-relevant information across the network hierarchy. In the pooled nuisance run, however, the same analysis preferentially recovered nuisance condition. The contrast is important because it shows that the framework does not merely declare neural network clusters to be informative or uninformative. Rather, it identifies which target variable is preserved by a given partition of activation space. This is relevant to broader work using deep networks as models of visual representation and as comparison systems for biological object recognition [26,27,29,30]. The result also clarifies the role of target specification. If the relevant target is a nuisance condition, the deeper ResNet-50 category maps in the pooled run are highly informative. If the relevant target is object class, superordinate animal–vehicle class, or an affordance-like grouping, the same maps are much less informative. Conversely, when nuisance variation is removed in the clean-only run, final-layer maps preserve substantially more object-relevant information. Therefore, the usefulness of a categorisation cannot be inferred from entropy or clustering structure alone. It depends on the relation between the category map and the variable that the system is required to preserve. The neural network result should not be interpreted as evidence that ResNet-50 lacks object information. The clean-only analysis shows the opposite. Rather, the pooled nuisance result shows that an unsupervised clustering procedure applied to transformed images can preferentially recover the dominant variance structure of the activation space. Under the present stimulus construction, this dominant structure is nuisance-related. This interpretation is consistent with concerns that neural-network representations can be strongly shaped by image statistics, texture, or transformation structure, rather than by semantic object category alone [25,32].

The conditional and supervised-readout analyses refine this interpretation. Conditional object information asks whether object-class information remains within nuisance conditions, whereas supervised readout asks which target information can be recovered by a decoder. These analyses therefore separate dominant unsupervised activation geometry from target-specific information availability. In the strong pooled-nuisance control, nuisance condition dominated the unsupervised category geometry, but object information remained partially available within nuisance conditions. In the mild-nuisance control, object-related information became more prominent across later layers, indicating that nuisance dominance was not an invariant property of the network representation. Rather, it depended on the strength of the imposed transformation structure. This result supports the target-relative interpretation of category maps. The same representational system can preserve multiple variables, but an unsupervised category map may organise most strongly around whichever variable is most salient in the activation geometry. Under strong transformation structure, nuisance condition can become the dominant preserved variable. Under milder nuisance variation, object-related structure becomes more visible. Under target-specific supervised training, the dominant decodable variable can shift toward the trained target. Thus, the relevant question is not simply whether a representation contains object information, but whether a particular compression or category map preserves the target variable needed for the task. This conclusion also connects the network analysis to a broader problem in cognition.

Object recognition is therefore not merely a problem of extracting object content; it is largely a problem of managing nuisance dimensions such as viewpoint, position, scale, texture, illumination, transformation, and context. This interpretation is consistent with neurophysiological work on the ventral visual stream, from early demonstrations of complex visual selectivity in macaque inferotemporal cortex to later work showing that object representations combine selectivity with tolerance to identity-preserving transformations [36,37,38,39,40]. The present controls suggest that nuisance handling is not a peripheral issue but part of the computational burden of forming useful category maps. A category map that is highly structured can still be functionally misaligned if it preserves nuisance variation more strongly than the behavioural or computational target.

Several limitations follow from the deliberately minimal design. The synthetic demonstrations use low-dimensional Gaussian stimulus spaces and explicitly defined target variables. This is useful for isolating the formal logic, but it does not capture the full structure of natural categories. Real categories involve multi-feature distributions, hierarchical dependencies, contextual variation, causal knowledge, embodiment, and task-specific constraints [2,41]. The present analysis should therefore be understood as a formal demonstration of a principle rather than a complete model of natural category learning. The neural network extension is also exploratory. CIFAR-10 images are small, and the nuisance transformations remain simplified relative to natural image variation [34]. Moreover, unsupervised clustering of activation vectors is only one way to derive category maps from a network. The conditional-information and supervised-readout analyses were therefore included as targeted controls rather than as an exhaustive analysis of network representations. The contrast between strong and mild nuisance conditions shows that the balance between object and nuisance preservation is sensitive to transformation strength. Further extensions could use representational similarity analysis, invariance metrics, alternative architectures, or downstream behavioural readouts [28,29]. Nevertheless, the result is informative because it reveals exactly the problem addressed by the manuscript: structured compression can preserve a variable that is not aligned with the intended target.

The framework also has biological relevance beyond the neural network demonstration. Behavioural and neural data can be analysed in the same terms by treating observed responses, neural population states, or model activations as candidate category maps. In biological systems, such maps need not be organised around the same target variable used by the experimenter. A partition shaped by one functional demand may nevertheless preserve information about another variable, thereby making an additional discrimination possible. This provides an information-theoretic way to evaluate the preconditions for functional reuse: before an existing representational or behavioural system can support a new target, that target must remain at least partly recoverable from the existing partition. In this restricted sense, the framework is compatible with ideas of pleiotropy and exaptive reuse, while keeping the inference close to the quantities estimated here [42,43,44].

The central implication is that category maps should be understood as target-preserving reductions of stimulus variation. Reduction alone is not sufficient. A useful category system preserves the information that matters for the organism, task, or model under consideration. Entropy describes the complexity of the induced category-label distribution. Mutual information with a specified target describes whether that category structure is useful.

## 5. Conclusions

The present study introduces a target-preservation view of categorisation. Its central claim is not simply that categories compress stimulus spaces, but that the value of a category map depends on which target variable remains informative after compression. This distinction is expressed quantitatively by separating category complexity, $H(C_{m})$, from target preservation, $I(C_{m};T)$, and $\mathrm{NMI}(C_{m},T)$. The synthetic demonstrations show that maps with similar complexity can differ sharply in the targets they preserve, that hierarchical category levels correspond to different target-dependent compressions, and that robustness under perturbation depends on map–target alignment. The neural-network extension shows that the same issue arises in learned representations: unsupervised activation-space partitions can preserve substantial information, but this information may concern nuisance conditions rather than object-relevant category structures. The additional network controls refine this conclusion. In the strong pooled-nuisance network analysis, nuisance condition dominated the unsupervised category geometry, but conditional object information remained detectable after nuisance condition was controlled. In the mild-nuisance control, object-related information became more prominent across later layers, showing that nuisance dominance depended on the strength of the imposed transformation structure rather than on an absence of object information in the network. The supervised-readout analysis further showed that the dominant decodable variable followed the training target: object-trained networks favoured object information, whereas nuisance-trained networks favoured nuisance information. Thus, the neural-network extension supports the central claim in a target-relative sense. A category map is not useful because it compresses or clusters a representation; it is useful to the extent that it preserves the target variable under the relevant nuisance structure.

Future implementations can extend the framework in several directions. First, supervised and unsupervised category maps can be compared across network architectures, training objectives, and task regimes. Second, multiple targets can be evaluated jointly, allowing category maps to be assessed not only for a single behavioural consequence but also for trade-offs among identity, action, reward, context, and nuisance tolerance. Third, the framework can be applied to empirical behavioural and neural data, where the target variable may be action selection, individual recognition, reward prediction, or context-dependent generalisation. Fourth, category learning can be modelled as an optimisation process in which agents search for maps that preserve target information under constraints on category complexity. Such an extension would connect the present analysis more directly to information-bottleneck approaches and computational models of category learning [11,16,17].

## Figures and Tables

**Table 1:** Overview of the five demonstrations. The table summarises the stimulus source, candidate category maps, target variables, main information-theoretic quantities, and purpose of each demonstration.

No.	Stimulus Source	Candidate Category Maps	Target Variables	Main Quantities	Purpose
1	Two-dimensional Gaussian stimulus space	Identity, action, geometric, nuisance, random, one-category	Y,A	$H(C_{m})$, $I(C_{m};Y)$, $I(C_{m};A)$, $Q_{T}$	Test whether compression alone determines usefulness.
2	Same flat stimulus space	Same candidate maps as Demonstration 1	Y,A,V,G	$\mathrm{NMI}(C_{m},T)$	Test whether usefulness depends on the selected target.
3	Perturbed flat stimulus space	Fixed identity, action, geometric, nuisance, random, and one-category classification rules	Y,A	$\mathrm{NMI}(C_{m},T)$ across perturbation levels	Test robustness of preserved information under perturbation.
4	Hierarchical two-dimensional Gaussian stimulus space	Subordinate, basic, action/superordinate-like, geometric, random, one-category	$Y_{\mathrm{sub}},Y_{\mathrm{basic}},A$	$H(C_{m})$, $I(C_{m};T)$, $\mathrm{NMI}(C_{m},T)$	Test category level as target-dependent compression.
5	CIFAR-10 images passed through pretrained ResNet-50	Layer-derived *k*-means category maps $C_{l,k}$	$Y_{\mathrm{obj}},Y_{\sup},A,V$	$\mathrm{NMI}(C_{l,k},T)$	Test whether learned activation-space partitions preserve object-relevant or nuisance-condition information.

**Table 2:** Candidate category maps used in the flat synthetic demonstrations.

Map	Category Variable	Definition
$m_{Y}$	$C_{m_{Y}}$	Identity-preserving map
$m_{A}$	$C_{m_{A}}$	Action-preserving map
$m_{G}$	$C_{m_{G}}$	Geometric map
$m_{V}$	$C_{m_{V}}$	Nuisance-preserving map
$m_{R}$	$C_{m_{R}}$	Random map
$m_{0}$	$C_{m_{0}}$	One-category collapsed map

**Table 3:** Representative robustness values for Demonstration 3. Values report normalised mutual information between the category assignment *C* and the relevant target variable under selected perturbation strengths. Identity-relevant preservation is reported as I(C;Y)/H(Y), and action-relevant preservation is reported as I(C;A)/H(A).

Target	Rule	Noise 0.0	Noise 0.4	Noise 1.0	Noise 2.2
Identity *Y*	Identity	0.979	0.927	0.646	0.235
Identity *Y*	Geometric	0.979	0.927	0.646	0.235
Identity *Y*	Action	0.561	0.530	0.375	0.140
Identity *Y*	Nuisance	0.000	0.000	0.000	0.000
Identity *Y*	Random	0.000	0.000	0.000	0.000
Identity *Y*	One category	0.000	0.000	0.000	0.000
Action *A*	Identity	0.973	0.920	0.652	0.243
Action *A*	Geometric	0.973	0.921	0.652	0.243
Action *A*	Action	0.972	0.918	0.648	0.239
Action *A*	Nuisance	0.001	0.001	0.001	0.001
Action *A*	Random	0.000	0.000	0.000	0.000
Action *A*	One category	0.000	0.000	0.000	0.000

**Table 4:** Representative quantitative values from the synthetic demonstrations. Values are computed from the category variable $C_{m}$ induced by map *m* and the indicated target variable *T*. Demonstration 1 shows aligned and misaligned flat-map contrasts, Demonstration 2 shows target dependence, and Demonstration 4 shows hierarchical target preservation.

Demo	Map	Target	$H(C_{m})$	$I(C_{m};T)$	$\mathrm{NMI}(C_{m},T)$
1	$m_{Y}$	*Y*	1.584	1.584	1.000
1	$m_{A}$	*A*	0.913	0.913	1.000
1	$m_{V}$	*Y*	1.585	0.001	<0.001
2	$m_{V}$	*V*	1.585	1.585	1.000
2	$m_{G}$	*G*	1.584	1.584	1.000
2	$m_{A}$	*Y*	0.913	0.913	0.576
4	$m_{\mathrm{sub}}$	$Y_{\mathrm{sub}}$	2.584	2.584	1.000
4	$m_{\mathrm{basic}}$	$Y_{\mathrm{basic}}$	1.584	1.584	1.000
4	$m_{\mathrm{act}}$	*A*	0.930	0.930	1.000
4	$m_{R}$	$Y_{\mathrm{sub}}$	2.584	0.008	0.003

**Table 5:** Exact k=10 values for the pooled-nuisance ResNet-50 run. The target columns report normalised mutual information between the layer-derived category map $C_{l,10}$ and each target variable; $Y_{\mathrm{obj}}$ denotes CIFAR-10 object class, $Y_{\sup}$ the animal–vehicle target, *A* the environment- or affordance-like target, and *V* nuisance condition.

Layer	$H(C_{l,10})$	$Y_{\mathrm{obj}}$	$Y_{\sup}$	*A*	*V*
activation_1_relu	3.118	0.116	0.237	0.134	0.004
activation_10_relu	3.232	0.082	0.198	0.093	0.901
activation_22_relu	3.176	0.093	0.185	0.142	0.975
activation_40_relu	3.151	0.105	0.197	0.131	0.903
activation_49_relu	2.789	0.137	0.253	0.119	0.837
avg_pool	3.088	0.173	0.344	0.143	0.894

## Data Availability

All data analysed in this study were either generated synthetically within the modelling pipeline or derived from the publicly available CIFAR-10 dataset processed through a pretrained ResNet-50 network. The MATLAB code used to construct the synthetic demonstrations, extract and analyse the layer-derived category maps, compute the information-theoretic summaries, and generate all figures is openly available at https://github.com/ChristophDahl/entropic-compression-category-maps under the MIT License. The repository includes a master script that reproduces the analyses and figures reported in this manuscript.
